# *CHD4* variants are associated with childhood idiopathic epilepsy with sinus arrhythmia

**DOI:** 10.1111/cns.13692

**Published:** 2021-06-09

**Authors:** Xiao‐Rong Liu, Ting‐Ting Ye, Wen‐Jun Zhang, Xuan Guo, Jie Wang, Shao‐Ping Huang, Long‐Shan Xie, Xing‐Wang Song, Wei‐Wen Deng, Bing‐Mei Li, Na He, Qian‐Yi Wu, Min‐Zhi Zhuang, Meng Xu, Yi‐Wu Shi, Tao Su, Yong‐Hong Yi, Wei‐Ping Liao, For the China Epilepsy Gene 1.0 Project

**Affiliations:** ^1^ Institute of Neuroscience and Department of Neurology of the Second Affiliated Hospital of Guangzhou Medical University Key Laboratory of Neurogenetics and Channelopathies of Guangdong Province and the Ministry of Education of China Guangzhou China; ^2^ Department of Pediatrics The Second Affiliated Hospital of Xi'an Jiao Tong University Xi'an China; ^3^ Epilepsy Center of Foshan First Hospital Foshan China

**Keywords:** *CHD4* gene, childhood idiopathic epilepsy, sinus arrhythmia

## Abstract

**Aims:**

*CHD4* gene, encoding chromodomain helicase DNA‐binding protein 4, is a vital gene for fetal development. In this study, we aimed to explore the association between *CHD4* variants and idiopathic epilepsy.

**Methods:**

Trios‐based whole‐exome sequencing was performed in a cohort of 482 patients with childhood idiopathic epilepsy. The Clinical Validity Framework of ClinGen and an evaluating method from five clinical‐genetic aspects were used to determine the association between *CHD4* variants and epilepsy.

**Results:**

Four novel heterozygous missense mutations in *CHD4*, including two de novo mutations (c.1597A>G/p.K533E and c.4936G>A/p.E1646K) and two inherited mutations with co‐segregation (c.856C>G/p.P286A and c.4977C>G/p.D1659E), were identified in four unrelated families with eight individuals affected. Seven affected individuals had sinus arrhythmia. From the molecular sub‐regional point of view, the missense mutations located in the central regions from SNF2‐like region to DUF1087 domain were associated with multisystem developmental disorders, while idiopathic epilepsy‐related mutations were outside this region. Strong evidence from ClinGen Clinical Validity Framework and evidences from four of the five clinical‐genetic aspects suggested an association between *CHD4* variants and epilepsy.

**Conclusions:**

*CHD4* was potentially a candidate pathogenic gene of childhood idiopathic epilepsy with arrhythmia. The molecular sub‐regional effect of *CHD4* mutations helped explaining the mechanisms underlying phenotypic variations.

## Graphical abstract

CHD4 missense mutations associated with multisystem developmental abnormalities were clustered in the central region from SNF2‐like region to DUF 1087 domain, while the variants associated with other mild phenotypes were located outside this region. The potentially molecular sub‐regional effects of missense mutations may explain for the phenotypic severity of CHD4 mutations and would help understand the underlying mechanisms of phenotypic variation.

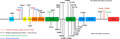

## 1 INTRODUCTION

*CHD4* gene (OMIM* 603277) encodes chromodomain helicase DNA‐binding protein 4 (CHD4), which belongs to the SNF2/RAD54 helicase family. It is an ATP‐dependent chromatin remodeler that is involved in epigenetic regulation of gene transcription, DNA repair, and cell cycle progression.^1^ *CHD4* is ubiquitously expressed across the whole lifespan, including brain. Homozygous knockout of *CHD4* in mice resulted in embryonic lethality with abnormal extraembryonic tissue physiology, absent blastocoele, and increased apoptosis. In humans, *CHD4* mutations were initially regarded to increase the risk of cancer.^2^ Lately, *CHD4* mutations have been reported to be associated with Sifrim‐Hitz‐Weiss syndrome (SIHIWES) (OMIM# 617159), which is an autosomal dominant intellectual developmental disorder with variable congenital defects involving cardiac, skeletal, and urogenital systems.^1^, ^3^ *CHD4* mutations have also been detected in the patients with neurodevelopmental disorders, Rett syndrome, schizophrenia, and pituitary stalk interruption syndrome, among whom rare cases presented epilepsy.^4^, ^5^, ^6^, ^7^ However, the role of *CHD4* in epilepsy remains largely unknown.

In this study, we performed trios‐based whole‐exome sequencing (WES) in a cohort of childhood epilepsy without acquired causes. Four novel *CHD4* mutations were identified in four unrelated families of childhood idiopathic epilepsy with sinus arrhythmia, suggesting that *CHD4* was potentially a candidate causative gene of epilepsy with arrhythmia. We further systematically reviewed all *CHD4* mutations and their corresponding phenotypes, aiming to explore the mechanisms underlying phenotypic variations.

## 2 MATERIALS AND METHODS

### 2.1 Subjects

A total of 482 patients with childhood unexplained epilepsy were recruited for genetic screening during January 2013–June 2020. Seizure and epilepsy were diagnosed and classified according to the proposals by International League Against Epilepsy.^8^, ^9^, ^10^, ^11^ he collected clinical data included semiology, evolution of the disorders, family histories, and the data of management. Video‐EEG recordings with ECG electrodes were obtained to confirm the diagnosis of epilepsy. Echocardiography was performed for the patients with the cardiac symptoms and/or arrhythmia during EEG monitoring. Brain magnetic resonance imaging (MRI) was conducted to exclude symptomatic epilepsy. Patients with acquired etiologies, such as tumor, trauma, infection, immunity, and stroke, were excluded. All the patients were followed up for at least one year.

The studies adhered to the guidelines of the International Committee of Medical Journal Editors with regard to patient consent for research or participation and received approval from the Ethics Committee of the Second Affiliated Hospital of Guangzhou Medical University. All participants provided written informed consents.

### 2.2 Identification of gene variants

The blood samples were obtained from the probands, their parents, and other available family members. Genomic DNA was extracted from peripheral blood using a QuickGene DNA whole blood kit (Fujifilm). Trios‐WES was conducted on the Illumina HiSeq 2500/4000 platform by BGI‐Shenzhen as previously reported.^12^ Population‐based filtration removed common variants presenting minor allele frequency ≥0.005 in the 1000 Genomes Projects, Exome Aggregation Consortium, and Genome Aggregation Database (gnomAD). Then, we retained potentially pathogenic variants containing frameshift, nonsense, canonical splice site, initiation codon, and missense mutations predicted as being damaging by in silico tools (http://varcards.biols.ac.cn/). The potential disease‐causing mutations in each case were screened under five models: epilepsy‐associated gene model, dominant/de novo model, autosomal recessive inheritance model, X‐linked model, and co‐segregation analysis model. After excluding the known epilepsy‐associated genes, the genes with de novo mutations, bi‐allelic mutations, hemizygous mutations, and mutations with segregations were selected for further studies to validate as possible novel epilepsy genes. The candidate variants were validated by Sanger sequencing. Potential pathogenic variants were predicted by multiple in silico prediction tools (http://www. varcards.biols.ac.cn/). Conservation of mutated positions was evaluated using sequence alignment of different species. All *CHD4* variants were annotated based on the transcript NM_001273.3.

### 2.3 Computational modeling

To evaluate the pathogenicity of candidate variants, the structure of *CHD4* was modeled to predict the effect of missense mutations on protein structure by using SWISS‐MODEL (https://swissmodel.expasy.org/). PyMOL 2.3 software was used for three‐dimensional protein structure visualization and analysis.

### 2.4 Statistical analysis

R statistical software (v3.4.1) was used for statistical analysis. The frequency of the *CHD4* variants in general population and in the cohort of childhood idiopathic epilepsy was compared by Fisher's exact test. A *p* value of < 0.05 was considered to be statistically significant.

### 2.5 Evaluating *CHD4* gene as a novel candidate epilepsy gene

To explore the genotype‐phenotype association, publications on *CHD4* mutations and related phenotypes were retrieved from the PubMed database till March 2020. Their molecular location and clinical data were systematically analyzed. We further used the Clinical Validity Framework that was developed by Clinical Genome Resource (ClinGen)^13^ to evaluate *CHD4* as a novel candidate epilepsy gene. We also evaluated epilepsy as a novel phenotype of *CHD4* variants from five clinical‐genetic aspects as we proposed recently.^14^

## 3 RESULTS

### 3.1 Identification of *CHD4* mutations

**FIGURE 1:**
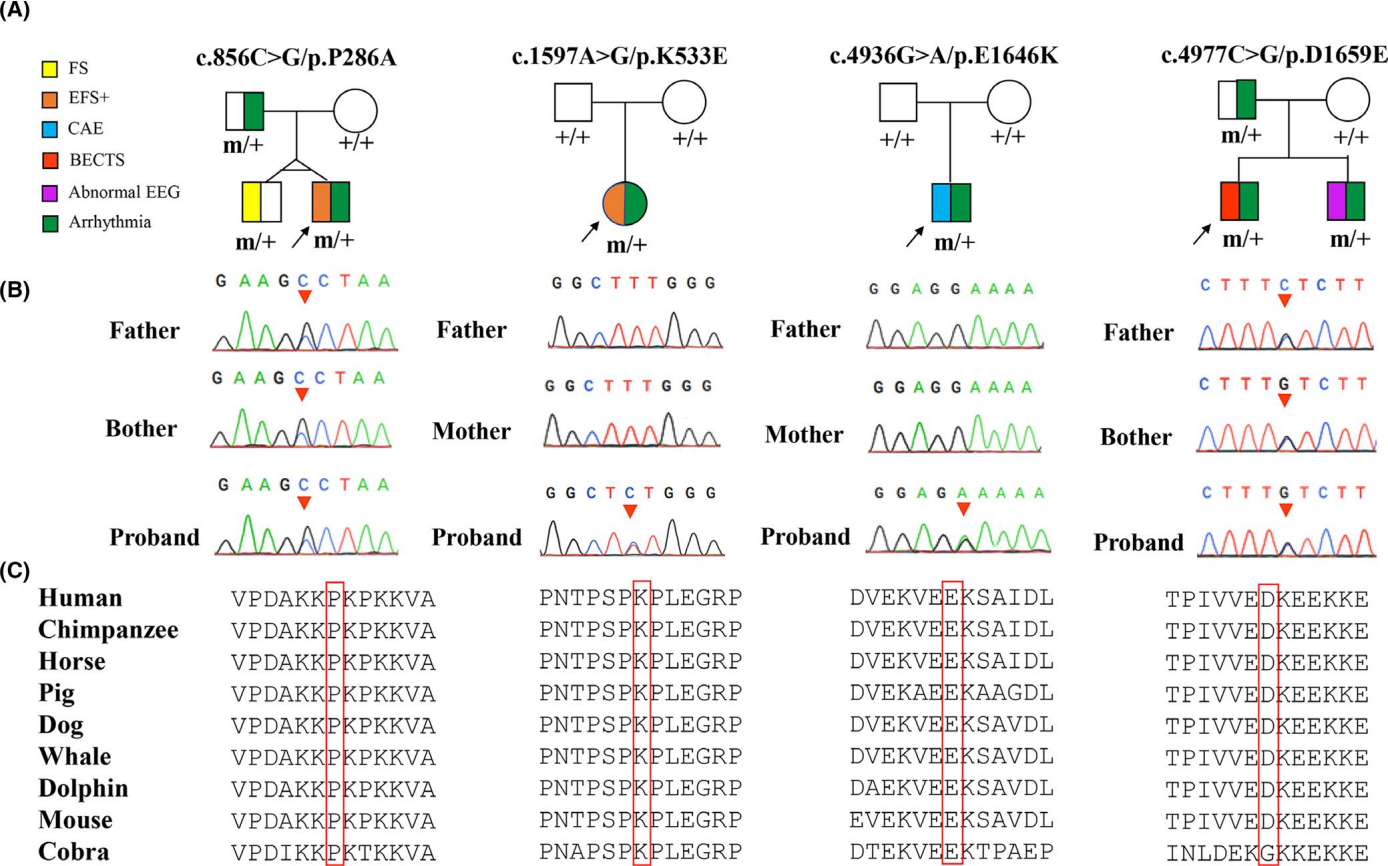
Genetic data on the patients with childhood idiopathic epilepsy with *CHD4* mutations. (A) Pedigrees of the four cases with *CHD4* mutations and their corresponding phenotypes. FS: febrile seizures; EFS+: epilepsy with febrile seizures; CAE: childhood absence epilepsy; BECTS: benign childhood epilepsy with centrotemporal EEG spikes. (B) DNA sequence chromatogram of the *CHD4* mutations. Arrows indicate the positions of the mutations. (C) The amino acid sequence alignment of the four missense mutations shows that the residues are highly conserved across vertebrates

Four novel heterozygous missense *CHD4* mutations were identified in four unrelated families with childhood idiopathic epilepsy, including two de novo mutations (c.1597A>G/p.K533E and c.4936G>A/p.E1646K) and two inherited mutations with co‐segregation within the families (c.856C>G/p.P286A and c.4977C>G/p.D1659E) (Figure 1A and 1B). The amino acid sequence alignment showed that all the four residues were highly conserved across higher vertebrates (Figure 1C). These mutations were predicted to be damaging by multiple in silico prediction tools (Table S1). All cases had no other pathogenic or likely pathogenic mutations in the 977 genes known to be associated with seizure disorders.^15^

Mutation P286A had a low allele frequency (4.09 × 10^−6^ in general population and 5.567 × 10^−5^ in East Asia population) in gnomAD database, but did not present in the control populations of gnomAD. The other three mutations (K533E, E1646K, and D1659E) did not appear in gnomAD database) (Table S1). A statistical analysis on the frequency of the *CHD4* variants showed a significant difference between the present cohort and the populations in gnomAD (general population, East Asian population, or controls in gnomAD (4/964 vs. 1/244520 in all populations of gnomAD, *p* = 1.222 × 10^−9^; vs. 1/17964 in East Asian population of gnomAD, *p* = 3.322 × 10^−5^) (Table S2).

### 3.2 Clinical features of the cases with *CHD4* mutations

**FIGURE 2:**
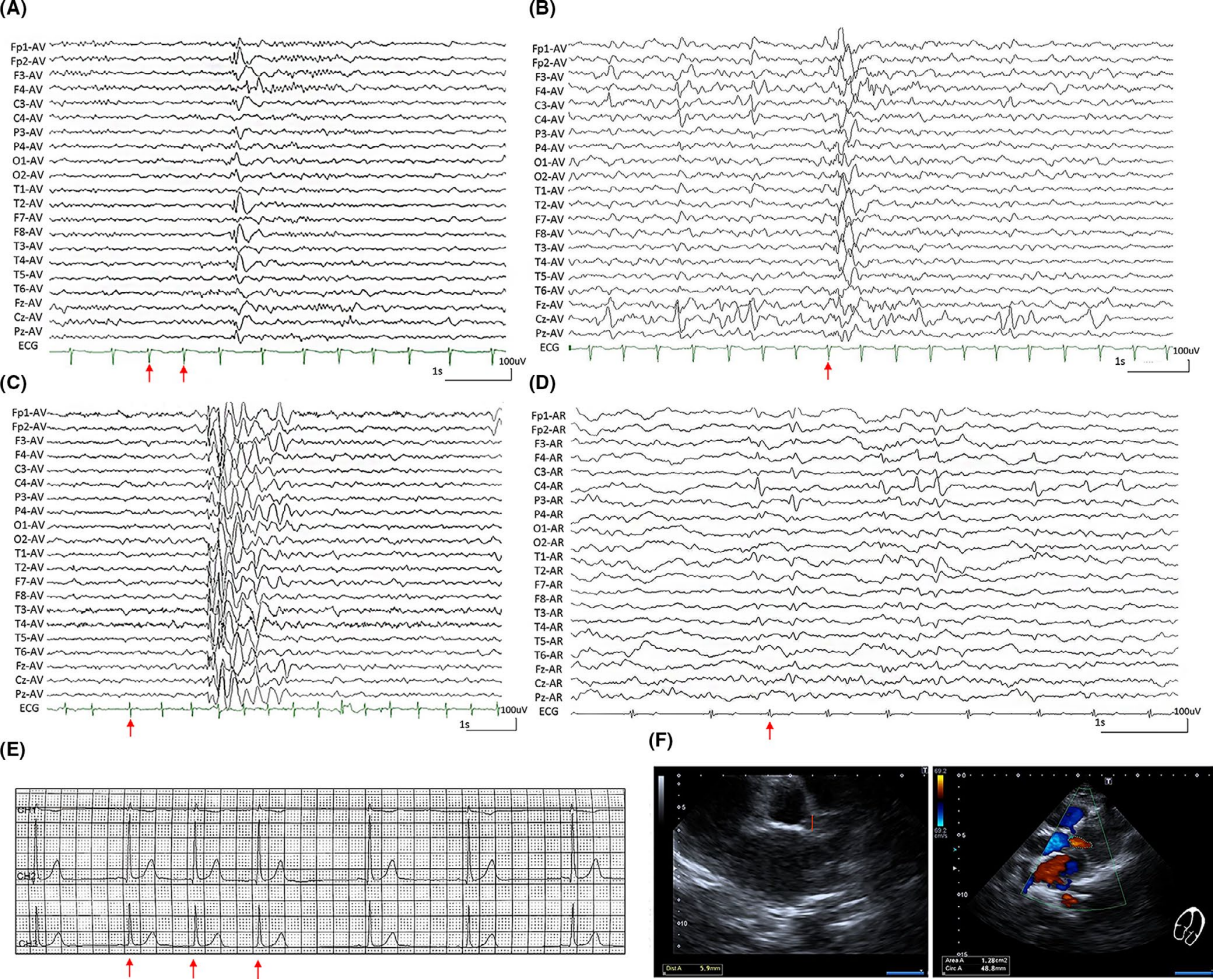
Changes in ECG, color Doppler ultrasound, and interictal EEG in the cases with *CHD4* mutations. (A and B) Interictal EEG of case 2 showed right frontotemporal spike and slow waves (A) and irregular generalized slow waves (B) with sinus arrhythmia (obtained at the age of 9 years). (C) Interictal EEG of case 3 showed intermittent high‐voltage 3 Hz spike and slow waves with sinus arrhythmia (obtained at the age of 6 years and after valproate treatment). (D) Interictal EEG of case 4 showed bilateral controtemportal spike‐slow waves with sinus arrhythmia (obtained at the age of 8 years). The arrows index the sinus premature beats during the ECG and video‐EEG monitoring. (E) ECG of case 1 showed distinct sinus arrhythmia (obtained at the age of 10 years). (F) Color Doppler ultrasound of case 1 showed mild aortic valve regurgitation (obtained at the age of 11 years)

The *CHD4* mutations were identified in four families with 8 individuals affected. The detailed clinical manifestations were summarized in Table 1. The median seizure‐onset age of the probands was 4 years old (ranging from 4 months to 8 years). The last follow‐up age ranged from 9 years to 15 years. All the probands had childhood idiopathic epilepsy characterized by shifting or bilateral focal discharges, or generalized spike‐slow waves with normal backgrounds of EEGs (Figure 2A–D). The probands in case 1 and case 2 had febrile seizures at the age of 4 months and 2 years, respectively, then followed by afebrile seizures. They were diagnosed as epilepsy with febrile seizure plus (EFS+). Case 3 was diagnosed as childhood absence epilepsy (CAE), and the proband of case 4 was a patient of benign childhood epilepsy with centrotemporal spikes (BECTS). Except case 3 who had daily typical absence seizures, the other three probands had infrequent complex partial seizures or secondary generalized tonic‐clonic seizures. Sinus arrhythmia with atrial premature was observed in all affected individuals (Figure 2E), except the brother of case 1 whose ECG was not available. Mild aortic valve regurgitation was detected in case 1 (Figure 2F). Brain MRI was normal in the four cases. All patients had normal intelligence and development, except the proband of case 4 presented borderline intelligence (Wechsler Intelligence Scale 74). They all became seizure‐free for at least one year on the routine dose of lamotrigine and/or sodium valproate.

**TABLE 1:** Clinical Features of the Individuals with *CHD4* Mutations

Case	Variants (NM_001273.3)	Diagnosis	Gender	Age	Seizure onset	Seizure course	EEG	Sinus arrhythmia	Brain MRI	Development	Effective AEDs	Prognosis	MAF in GnomAD	ACMG
Case 1–1	c.856C>G/p.P286A	EFS+	Male	11 years	FS at 4 months; CPS at 6 years	FS twice before 2 years; CPS and sGTCS twice /year for 4 years	Normal	+	Normal	Normal	VPA	Sz free 1 year	4.09 × 10^−6^ (0 in controls)	US (PP1+PP3)
Case 1–2 (B)	c.856C>G/p.P286A	FS	Male	11 years	2 years	FS once at 2 years	NA	NA	Normal	Normal	‐	Sz free 9 years		
Case 1–3 (F)	c.856C>G/p.P286A	‐	Male	46 years	‐	‐	NA	+	NA	Normal	‐	‐		
Case 2	c.1597A>G/p.K533E	EFS+	Male	10 years	Febrile and afebrile GTCS at 2 years	GTCS 5–6 times/year for 4 years	Right frontotemporal spike‐slow wave and generalized slow waves	+	Normal	Normal	VPA	Sz free 4 years	‐	LP (PS2+PM2+PP3)
Case 3	c.4936G>A/p.E1646K	CAE	Female	9 years	6 years	GTCS 2 times/year and absence daily for 1 year	Generalized 3 Hz spike‐slow waves	+	Normal	Normal	LTG 25 mg bid	Sz free 3 years	‐	LP (PS2+PM2+PP3)
Case 4–1	c.4977C>G/p.D1659E	BECTS	Male	15 years	8 years	GTCS 1–2 times/year for 2 years	Bilateral controtemportal spike‐slow waves	+	Normal	Borderline	LTG 62.5 mg bid, VPA 0.25 bid	Sz free 5 years	‐	US (PM2+PP1+PP3)
Case 4–2 (B)	c.4977C>G/p.D1659E	‐	Male	15 years	‐	‐	Generalized spike‐slow waves	+	NA	Normal	‐	‐		
Case 4–3 (F)	c.4977C>G/p.D1659E	‐	Male	45 years	‐	‐	NA	+	NA	Borderline	‐	‐		

Abbreviations: AEDs, antiepileptic drugs; B, brother; BECTS, benign childhood epilepsy with centrotemporal EEG spikes; CAE, childhood absence epilepsy; CPS, complex partial seizure; EEG, electroencephalogram; EFS+, epilepsy with febrile seizure plus; F, father; FS, febrile seizure; GTCS, generalized tonic‐clonic seizure; LP, likely pathogenesis; LTG, lamotrigine; MAF, minor allele frequency from gnomAD; MRI, magnetic resonance imaging; NA, not available; sGTCS, secondary generalized tonic‐clonic seizure; Sz, seizure; US, uncertain significant; VPA, valproate.

### 3.3 Structural alteration of *CHD4* protein and genotype‐phenotype correlation of *CHD4* variants

**FIGURE 3:**
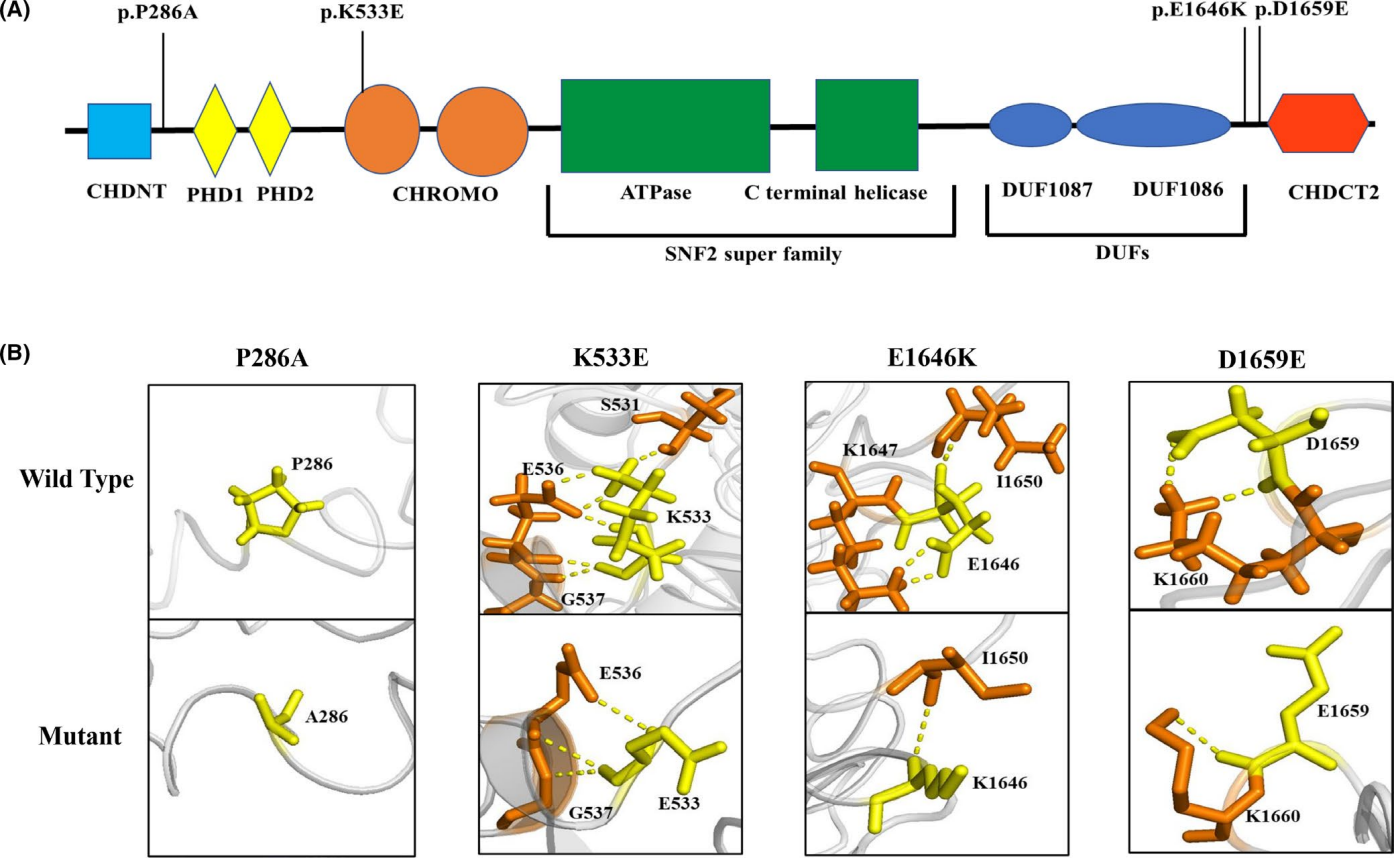
Schematic illustration of mutation and interactions with surrounding amino acids. (A) Schematic diagram of *CHD4* and the localization of the *CHD4* mutations identified in this study. (B) Hydrogen bond changes in mutants P286, K533, E1646, and D1659

As shown schematically in Figure 3, *CHD4* successively contains CHD‐N‐terminal domain (CHDNT), two plant homeodomain (PHD) zinc fingers, tandem chromatin organization modifier domain (CHROMO), ATPase/helicase domains, domains of unknown function (DUFs), and CHD c‐terminal domain 2 (CHDCT2). Mutation P286A was located in the liker between CHDNT and PHD1; mutation K533E was located in CHROMO, and mutations E1646K and D1659E were located between DUF1086 and CHDCT2 (Figure 3A).

The molecular effect of the missense variants was further analyzed by protein modeling using SWISS‐MODEL. At residue P286, no hydrogen bond with the surrounding residues was formed in both wild‐type and the mutant residue A286 (Figure 3B). Residue K533 was originally formed four hydrogen bonds with E536, and one with G537 and S531 each. When lysine was replaced by glutamic acid at 533, the hydrogen bonds with S531 and two hydrogen bonds with E536 were destroyed. Residue E1646 originally formed two hydrogen bonds with residue K1647 and one hydrogen bond with I1650. Contrastingly, when the glutamic acid was replaced by lysine at 1646, the two hydrogen bonds with residue K1647 were destroyed. Residue D1659 originally formed two hydrogen bonds with residue K1660. When aspartic acid was replaced by glutamic acid at 1659, only one hydrogen bond was kept.

To explore the mechanism underlying phenotypic variations, we analyzed genotype‐phenotype relations in all reported *CHD4* mutations. To date, 51 mutations have been reported, including 44 missense and 7 destructive mutations (3 nonsense, 1 frameshift, 2 small deletion, and 1 small insertion mutations). ^1^, ^2^, ^3^, ^4^, ^5^, ^6^, ^7^, ^16^, ^17^, ^18^, ^19^, ^20^, ^21^, ^22^, ^23^, ^24^, ^25^, ^26^, ^27^ Their clinical and molecular details were listed in Table S3. All the destructive mutations were associated with SIHIWES, while the phenotypes of the missense mutations varied from mild phenotypes such as pituitary stalk interruption syndrome with incomplete penetrance and schizophrenia without developmental disorders, to severe multisystem development abnormalities.

**FIGURE 4:**
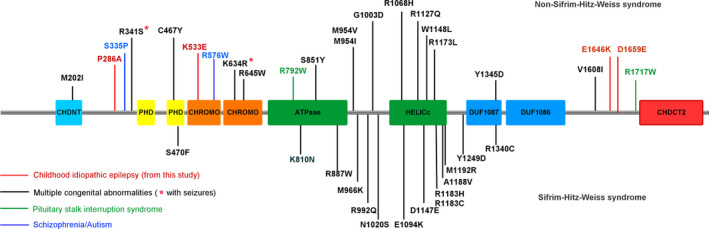
Schematic diagram of missense *CHD4* mutations and their locations on *CHD4*. Locations of the missense mutations with explicit phenotypes were indicated on the structure of CHD4. Mutations associated with non‐Sifrim‐Hitz‐Weiss syndrome were shown at the top of the structural diagram. Mutations associated with Sifrim‐Hitz‐Weiss syndrome were shown at the bottom

As our previous studies showed that molecular sub‐regional location of the missense mutations was a critical factor to determine the pathogenicity of variants and associated with phenotypic severity,^14^, ^28^ we analyzed the locations of missense mutations on *CHD4* protein. Among the 35 missense mutations with detailed phenotypes, 27 mutations were associated with multisystem developmental disorders, including 13 mutations associated with full SIHIWES and 14 mutations associated with developmental disorders in more than one systems but not meet the diagnosis of SIHIWES. Majority of these mutations associated with multiple congenital abnormalities (20/27, 74.1%) clustered in the central region from SNF2 region to DUF1087 domain (specifically from residue 810 to 1345), while mutations with other phenotypes (including autism spectrum disorder, schizophrenia, pituitary stalk interruption syndrome, and epilepsy) were exclusively located outside this region (Figure 4), suggesting a molecular sub‐regional effect of *CHD4* mutations. The six seizure‐related mutations, including the two previously reported mutations, were also located outside the region from residue 810 to 1345, in CHDNT‐PHD1 linker (P286A and R341S), CHROMO (K533EA and K634R), and DUF1086‐CHDCT2 linker (E1646K and D1659E), respectively.

### 3.4 Evaluation of epilepsy as a novel phenotype of *CHD4* variants

We evaluated the *CHD4* variant epilepsy correlation using ClinGen Clinical Validity Framework. The total allowable points for genetic aspect and experimental aspect were 6.3 and 6, respectively. The results of clinical validity summary matrix were 12.3 points that was categorized as “Strong,” supporting the association between *CHD4* variants and epilepsy (Table 2). In the five clinical‐genetic dimensional evaluation, evidence from four of the five clinical‐genetic aspects (repetition, genotype‐phenotype correlation, inheritance pattern, and molecular sub‐regional implications) was ranked “Yes,” suggesting that epilepsy was a novel phenotype of *CHD4* variants (Table S4). These findings indicated that *CHD4* mutations are potentially a candidate pathogenic gene of epilepsy.

**TABLE 2:** Evaluating the Clinical Validity of *CHD4*‐Epilepsy Associations Based on the Framework Developed by the Clinical Genome Resource

	Evidence type		Case information			Suggested points/Case		Points given	Max score
						Default	Range		
Case‐level data	Variant evidence	Autosomal dominant OR X‐linked disorder	Variant is de novo			2	0–3	4^a^	12
			Proband with predicted or proven null variant			1.5	0–2	0	10
			Proband with other variant type with some evidence of gene impact			0.5	0–1.5	2.3^b^	7
		Autosomal recessive	Two variants in trans and at least one de novo or a predicted/proven null variant			2	0.3	‐	12
			Two variants (not predicted/proven null) with some evidence of gene impact in trans			1	0–1.5	‐	
	Segregation evidence		Evidence of segregation in one or more families	LOD Score Example	3	5	0–7	0	7
					2	4			
					1.5	3			
					1	0.5			
Case‐control data	Case‐control study type		Case‐control quality criteria			Suggested points/Study		Points given	Max score
	Single variant analysis		Variant Detection Methodology Power Bias and Confounding Factors Statistical Significance			0–6		‐	12
	Aggregate variant analysis					0–6		6^c^	
Total allowable points for genetic evidence								6.3	12

Evidence category	Evidence type	Suggested points		Points given	Max score
		Default	Range		
Function	Biochemical function	0.5	0–2	0.5^d^	2
	Protein interaction		0–2	0.5^e^	
	Expression		0–2	0.5^f^	
Functional alteration	Cells from affected individual	1	0–2	0	2
	Engineered cells	0.5	0–1	0.5^g^	
Models & rescue	Animal model	2	0–4	3^h^	4
	Cell culture model system	1	0–2	1^i^	
	Rescue in animal model	2	0–4	0	
	Rescue in engineered equivalent	1	0–2	0	
Total allowable points for experimental evidence				6	6
Clinical validity summary matrix				12.3	Strong

^a^ Two de novo missense variants. (2 pts/case × 2 cases).

^b^ The four variants in the present study were all low MAF. Three of four variants did not appear in gnomAD database. (0.1 pts/case × 4 cases). The four variants were predicted damaging by multiple in silico programs. (0.4 pts/case × 4 cases). Three variants were predicted to change hydrogen bonds based on 3D structure. (0.1 pts/case × 3cases).

^c^ This is an aggregate analysis. Comparing to allele number in gnomAD‐control populations and in controls of gnomAD‐East Asian populations, the frequency of the variants in the present cohort is significant higher (Table S2). (Assigned 6 pts.). The points are not included in total allowable points for genetic evidence.

^d^ CHD4 protein belongs to the SNF2/RAD54 helicase family, and acts as one of the motor subunit of the NuRD complex (nucleosome remodeling and deacetylase activities) that is one of the major transcriptional repressors influencing different physiological conditions or different cell/tissue types. Several members of the SNF2/RAD54 helicase family (CHD2, CHD3, and CHD7) were associated with epilepsy and neurodevelopmental disorders. (Assigned 0.5 pts.).

^e^ CHD4 protein is one of the motor subunits of the NuRD complex, in which CHD4 protein combines with GATAD2B. The mutations in *GATAD2B* gene were associated with epilepsy (Shieh C, 2020. PMID: 31949314). (Assigned 0.5 pts.).

^f^ CHD4 gene is ubiquitously expressed across the whole lifespan, including brain. (Assigned 0.5 pts.).

^g^ Alterations in ATP hydrolysis and chromatin remodeling activities were observed in *CHD4* mutant HEK293 cells. (Weiss K, 2019. PMID: 31388190) (Assigned 0.5pts.).

^h^ Mice homozygous for *CHD4* null alleles exhibited embryonic lethality before implantation, abnormal extraembryonic tissue physiology, absent blastocoele, and increased apoptosis. (MGI:1344380). (Assigned 3 pts.).

^i^ In mammalian cells, CHD4 exhibited strong nuclear localization patterns, and rapidly recruited to DNA repairs sites, which revealed a subset of specific target genes (Hoffmeister H, 2017. PMID: 28977666). (Assigned 1 pt.).

## 4 DISCUSSION

*CHD4* gene is located at chromosomal locus 12p13.31 covering approximately 500 kb of genomic DNA. *CHD4* protein, also known as Mi‐2β, is the catalytic core subunit of the nucleosome remodeling and deacetylation (NuRD) complex. Experimentally, *CHD4* is one of essential proteins for numerous developmental events, including ensuring proper timing of the switch from stem cell lineages to differentiated cell types, maintaining cell differentiation, and activating DNA damage response pathways.^29^ Homozygous *CHD4* knockout mice have exhibited abnormal cell cycle and even preweaning lethality with complete penetrance. Clinically, the majority of *CHD4* mutations were associated with varying degrees of multiple congenital anomalies (heart, skeleton, brain, etc).^21^ These evidences indicate that *CHD4* is essential for normal development in higher organisms.^29^, ^30^, ^31^, ^32^, ^33^, ^34^ In the central nervous system, *CHD4* promotes the early proliferation of progenitors during cerebral cortical development. *CHD4* mutations were identified in the patients with multiple neurological disorders, such as neurodevelopmental delay, cognitive disability, and autism, suggesting *CHD4* protein plays a crucial role in neurodevelopment. However, the association between *CHD4* mutations and epilepsy remains unknown. In the present study, four novel missense *CHD4* mutations, including two de novo mutations and two inherited mutations with co‐segregation in families, were identified in the patients with childhood idiopathic epilepsies and sinus arrhythmia. All mutations present no or very low allele frequencies in the gnomAD database and statistically higher frequency in the cohort of epilepsy than in the populations of gnomAD. The strong supporting evidence from Clinical Validity Framework of ClinGen and the evidence from four of five clinical‐genetic aspects suggest that *CHD4* mutations were potentially associated with childhood idiopathic epilepsies. *CHD4* gene was considered as a candidate pathogenic gene of epilepsy.

CHD family proteins are characterized by a SNF2‐like helicase‐ATPase domain located in the central region and signature tandem chromodomains located in the amino‐terminal region, playing vital roles in the maintenance, survival, or proliferation of stem cell populations and in directing cell fate decisions of their progeny.^35^ The family comprises nine proteins (CHD1‐9), which were divided into three subfamilies based on domain homology (Li 2014). Subfamily I members (CHD1 and CHD2) contain a DNA‐binding domain with a preference for binding ATP‐rich sequences. Subfamily II members (CHD3, *CHD4*, and CHD5) lack a defined DNA‐binding domain, but harbor dual plant homeodomain zinc finger motifs upstream of the chromodomains. Subfamily III members (CHD6, CHD7, CHD8, and CHD9) contain additional functional motifs in their C‐terminal regions.^36^ Specific CHD proteins form distinct complexes, exhibit unique functions and preferences for repressive or active histone marks, and regulate the differentiation of mesenchymal stem cells into distinct lineages.^35^ However, their functions and combining substrates are overlapping.^36^ All CHD family genes were associated with neurodevelopmental disorders, while CHD1/2/3/7/8 genes were also implicated in epilepsies or seizures.^37^, ^38^ The patients with mutations in *CHD1*/*3*/*7*/*8* genes had epilepsy as a concomitant symptom of developmental abnormalities, while *CHD2* mutations caused epileptic encephalopathy as a core phenotype. In this study, *CHD4* mutations were identified in the patients with childhood idiopathic epilepsy characterized by infrequent seizures, normal background in EEG, normal neurodevelopment, and favorable outcome, indicating that *CHD4* was a candidate causative gene of childhood idiopathic epilepsy. However, the mechanism of phenotypic variation caused by CHD family genes is unclear. From the remodeling mechanisms/biological functions point of view, CHD2 acts as a monomer and is associated with relatively pure phenotype. On the contrary, *CHD4* performs functions as one part of NuRD complexes, potentially explaining phenotypes of more complex clinical symptoms. Similarly, CHD7 combines with polybromo‐ and BRG1‐associated factor‐containing complex^29^, ^39^ and is associated with complex phenotypic spectrum including CHARGE syndrome.^40^

In previous studies, *CHD4* mutations were associated with severe multisystem developmental abnormalities mostly, while in this study, the mutations were identified in patients with idiopathic childhood epilepsy. Further analysis showed that the *CHD4* missense mutations associated with multisystem developmental abnormalities were clustered in the central region from SNF2‐like region to DUF 1087 domain, while the variants associated with other mild phenotypes were located outside this region, suggesting a molecular sub‐regional effect. Moreover, seizure‐related mutations were located in CHNDT‐PHD1linker, CHROMO domain, and DUF1086‐CHDCT2 linker, suggesting the mutations in these regions were potentially related to increased susceptibility of epilepsy.

In this study, the seizures in all patients occurred in childhood, which was consistent with increased *CHD4* expression in this stage. Clinically, it is common that CAE and BECTS consecutively or contemporarily coexist in the same patients,^41^ while 11.8% children with CAE and 45.6% children with BECTS had family or personal history of febrile seizures.^42^, ^43^, ^44^ In this study, children with *CHD4* mutations presented as CAE, BECTS, and EFS+, similar as several previously reported genes associated with idiopathic epilepsy, such as GABRG2, SCN9A, and GRIN2A,^45^, ^46^ suggesting these epileptic syndromes potentially have similar etiologies.

*CHD4* gene is also expressed in heart at a middle level. In this study, seven affected individuals had asymptomatic sinus arrhythmia. One proband had mild cardiac structural anomalies without cardiac symptom. Previously, 65% of reported cases with *CHD4* mutations had heart defects, including atrial septal defect, ventricula septal defect, pulmonary stenosis/anomaly, patent ductus arteriosus, tetralogy of fallot, mitral valve anomaly, ebstein anomaly, and truncus arteriosus.^21^ Several genes related to both epilepsy and cardiac disorders present risk of sudden unexpected death in epilepsy, which is the leading cause of epilepsy‐related premature mortality.^47^, ^48^, ^49^ Thus, special attention should be paid to the patients with *CHD4* mutations, who may present not only benign epilepsies such as BECTS/CAE but also potentially had sinus arrhythmia, which requires specialized health consultation and extra follow‐up. Further investigations are required to determine the risk of cardiogenic sudden death caused by *CHD4* mutations.

In conclusion, this study identified four novel missense mutations in the patients with childhood idiopathic epilepsy with sinus arrhythmia. These mutations presented significantly higher frequent in case cohort than general populations. The strong supporting evidence from Clinical Validity Framework of ClinGen and evidence from four of five clinical‐genetic aspects suggested the association between *CHD4* variants and epilepsy, suggesting that *CHD4* mutations are potentially a candidate pathogenic gene of epilepsy with sinus arrhythmia. The potentially molecular sub‐regional effect of missense mutations would help understanding the underlying mechanisms of phenotypic variation.

## CONFLICT OF INTEREST

All authors claim that there are no conflicts of interest.

## Supporting information

Table S1

Table S2

Table S3

Table S4

## Data Availability

The data that support the findings of this study are available from the corresponding author upon reasonable request.
