# Supplementary material for: CHD4 variants are associated with childhood idiopathic epilepsy with sinus arrhythmia
Source: CNS Neurosci Ther. 2021 Jun 9;27(10):1146–56. doi: 10.1111/cns.13692 (PMC8446219; doi:10.1111/cns.13692)
Supplement: Supplementary file 1 — Table S1 [file CNS-27-1146-s002.doc]

| **Case** | **Coordinate (hg19)** | **Variants**  **(NM_001273.3)** | **Inheritance** | **MAF** | **MAF-East Asian** | **CADD** | **ClinPred** | **Fathmm-MKL** | **FitCons** | **GERP++** | **Mutation-Taster** | **PhastCons** | **Polyphen2** |
| --- | --- | --- | --- | --- | --- | --- | --- | --- | --- | --- | --- | --- | --- |
| Case 1 | chr12: 6710163 | c.856C>G  p.P286A | segregated with father | 4.09×10^-6^  (0 in controls) | 5.567×10^-5^  (0 in controls) | damaging (22.8) | pathogenic (0.93966233) | damaging (0.988) | damaging (0.707) | conserved (3.91) | disease-causing (1) | conserved (1.000) | probably-  damaging (1.0) |
| Case 2 | chr12: 6707477 | c.1597A>G  p.K533E | de novo | - | - | damaging (23.6) | pathogenic (0.83186787) | damaging (0.964) | damaging (0.707) | conserved (3.81) | polymorphism (0.681) | conserved (1.000) | benign (0.387) |
| Case 3 | chr12: 6688057 | c.4936G>A  p.E1646K | de novo | - | - | damaging (23.5) | pathogenic (0.73000252) | damaging (0.900) | damaging (0.707) | conserved (5.7) | disease-causing (1.000) | conserved (1.000) | possibly-  damaging (0.59) |
| Case 4 | chr12: 6688016 | c.4977C>G  p.D1659E | segregated with father | - | - | tolerable (12.18) | benign (0.07821534) | damaging (0.644) | damaging (0.707) | conserved (3.88) | polymorphism (0.843) | conserved (1.000) | benign (0.0) |

**Supplementary Table 1.** **Genetic Features of the *CHD4* Mutations**

**Abbreviations:** MAF, minor allele frequency from gnomAD.
