# Supplementary material for: CHD4 variants are associated with childhood idiopathic epilepsy with sinus arrhythmia
Source: CNS Neurosci Ther. 2021 Jun 9;27(10):1146–56. doi: 10.1111/cns.13692 (PMC8446219; doi:10.1111/cns.13692)
Supplement: Supplementary file 2 — Table S2 [file CNS-27-1146-s004.docx]

**Supplementary Table 2. Gene-based Burden Analysis for *CHD4* Mutations Identified in This Study**

|  | **Allele count/number in this study** | **Allele count/number in gnomAD-all populations** | **Allele count/number in gnomAD-East Asian populations** | **Allele count/number in controls of gnomAD-all populations** | **Allele count/number in controls of gnomAD-East Asian populations** |
| --- | --- | --- | --- | --- | --- |
| **Identified *CHD4* mutations** |  |  |  |  |  |
| chr12: 6710163: c.856C>G/p.P286A | 1/964 (0.00104) | 1/244520 (0.000004090) | 1/17964 (0.00005567) | 0/106590 | 0/9038 |
| chr12: 6707477: c.1597A>G/p.K533E | 1/964 (0.00104) | –/– | –/– | –/– |  |
| chr12: 6688057: c.4936G>A/p.E1646K | 1/964 (0.00104) | –/– | –/– | –/– |  |
| chr12: 6688016: c.4977C>G/p.D1659E | 1/964 (0.00104) | –/– | –/– | –/– |  |
| **Total** | 4/964 (0.00415) | 1/244520 (0.000004090) | 1/17964 (0.00005567) | 0/106590(0) | 0/9038(0) |
| ***p* value** |  | 1.222×10^-9^ | 3.322×10^-5^ | 6.52×10^-9^ | 8.71×10^-5^ |
| **OR (95% CI)** |  | 1030.082(99.69482-4.503600×10^15^) | 73.96426(7.322704-3561.047927) | Inf (72.85125-Inf) | Inf (6.176248-Inf) |

*p* values and odds ratio were estimated with 2-sided Fisher’s exact test.

Abbreviations: CI, confidence interval; gnomAD, Genome Aggregation Database; OR, odd ratio.
