# Supplementary material for: CHD4 variants are associated with childhood idiopathic epilepsy with sinus arrhythmia
Source: CNS Neurosci Ther. 2021 Jun 9;27(10):1146–56. doi: 10.1111/cns.13692 (PMC8446219; doi:10.1111/cns.13692)
Supplement: Supplementary file 3 — Table S3 [file CNS-27-1146-s003.docx]

| **Supplementary Table 3. Summary of Clinical Features in the Cases with *CHD4* Mutations** | | | | | | | | | | | | | |  |
| --- | --- | --- | --- | --- | --- | --- | --- | --- | --- | --- | --- | --- | --- | --- |
|  |  |  | **Domain** | | **Phenotype** | | **Intellectual disability** | **Neurodevelopmental**  **disorders** | **Seizure** | **Skeletal**  **anomalies** | **Heart anomalies** | **Hearing loss** | **Reference** | |
|  | c.420T>G/p.D140E | NA | N-CHDNT linker | Cancer, increased risk, association with | | | NA | NA | NA | NA | NA | NA | Yamada (2015) | |
|  | c.606G>A/p.M202I | De novo | CHDNT | Conotruncal heart defects with extracardiac anomalies | | | NA | + | NA | - | + | - | Jin (2017) | |
|  | c.835C>T/p.R279C | De novo | CHDNT-PHD1 | Intellectual disability | | | + | NA | NA | NA | NA | NA | Baker (2019) | |
|  | c.838G>A/p.V280I | NA | CHDNT-PHD1 | Neurodevelopmental disorder | | | + | NA | NA | NA | NA | NA | Trinh (2019) | |
|  | c.856C>G/p.P286A | Father | CHDNT-PHD1 | EFS+ | | | - | - | + | - | + | - | Present study | |
|  | c.1003T>C/p.S335P | De novo | CHDNT-PHD1 | Autism spectrum disorder | | | NA | NA | NA | NA | NA | NA | Wang (2016) | |
|  | c.1021C>A/p.R341S | De novo | CHDNT-PHD1 | Epileptic encephalopathy | | | + | NA | + | NA | NA | NA | Li (2016) | |
|  | c.1340A>G/p.H447R | NA | PHD1-PHD2 | Neurodevelopmental disorder | | | + | NA | NA | NA | NA | NA | Trinh (2019) | |
|  | c.1400G>A/p.C467Y | De novo  De novo  De novo | PHD2 | Congenital heart disease  Intellectual disability/developmental delay  Developmental disorder | | | +  +  NA | +  +  + | NA  NA  NA | NA  NA  NA | +  NA  NA | NA  NA  NA | Sifrim (2016)  Kosmicki (2017)  McRae (2017) | |
|  | c.1409C>T/p.S470F | NA | PHD2 | Sifrim–Hitz–Weiss syndrome | | | + | + | NA | + | - | + | Weiss(2019) | |
|  | c.1597A>G/p.K533E | De novo | CHROMO | EFS+ | | | - | - | + | - | + | - | Present study | |
|  | c.1726C>T/p.R576W | De novo  De novo | CHROMO | Schizophrenia  Schizophrenia | | | NA  NA | NA  NA | NA  NA | NA  NA | NA  NA | NA  NA | Girard (2011)  Li (2016) | |
|  | c.1901A>G/p.K634R | De novo | CHROMO | Neurodevelopmental disorder | | | + | + | + | + | NA | NA | Trinh (2019) | |
|  | c.1933C>T/p.R645W | De novo | CHROMO | Developmental disorder | | | + | + | NA | + | NA | NA | McRae (2017) | |
|  | c.2143C>T/p.Q715X | De novo | CHROMO- ATPase | Developmental disorder | | | + | + | NA | NA | NA | NA | McRae (2017) | |
|  | c.2219_2221del/p.S740del | NA | ATPase | Sifrim–Hitz–Weiss syndrome | | | + | + | NA | + | NA | NA | Weiss (2019) | |
|  | c.2374C>T/p.R792W | Father | ATPase | Pituitary stalk interruption syndrome | | | NA | NA | NA | NA | NA | NA | Zwaveling-Soonawala (2018) | |
|  | c.2430G>T/p.K810N | NA | ATPase | Sifrim–Hitz–Weiss syndrome | | | + | + | NA | NA | + | - | Weiss (2019) | |
|  | c.2552C>A/p.S851Y | De novo  De novo | ATPase | Congenital heart disease  Developmental disorder | | | +  NA | +  + | NA  NA | NA  NA | +  NA | NA  NA | Sifrim (2016)  McRae (2017) | |
|  | c.2612T>C/p.I871T | De novo  De novo  De novo | ATPase | neurodevelopmental disorder  Autism spectrum disorder  Autism spectrum disorder | | | -  NA  NA | +  NA  NA | NA  NA  NA | NA  NA  NA | NA  NA  NA | NA  NA  NA | Kosmicki (2017)  Wang (2016)  Iossifov (2014) | |
|  | c.2629C>G/p.R877G | - | ATPase | Multiple congenital anomalies | | | NA | NA | NA | NA | NA | NA | Bruel (2019) | |
|  | c. 2659C>T/p. R887W | NA | ATPase | Sifrim–Hitz–Weiss syndrome | | | NA | + | NA | NA | + | NA | Weiss(2019) | |
|  | c.2662delC/p.V888YfsX2 | NA | ATPase | Sifrim–Hitz–Weiss syndrome | | | NA | + | NA | NA | - | - | Weiss(2019) | |
|  | c.2862G>T/p.M954I | De novo  De novo  De novo | ATPase-HELICc | Congenital heart disease with neurodevelopmental disability  Left ventricular obstruction with extracardiac anomalies  Congenital heart disease | | | +  +  NA | NA  NA  NA | NA  NA  NA | NA  NA  NA | +  +  + | NA  NA  NA | Homsy (2015)  Jin (2017)  Kosmicki (2017) | |
|  | c.2860A>G/p.M954V | De novo | ATPase-HELICc | Developmental disorder | | + | | + | NA | NA | + | NA | McRae (2017) | |
|  | c.2897T>A/p.M966K | NA | ATPase-HELICc | Sifrim–Hitz–Weiss syndrome | | NA | | + | NA | NA | + | NA | Weiss (2016) | |
|  | c.2975G>A/p.R992Q | NA | ATPase-HELICc | Sifrim–Hitz–Weiss syndrome | | + | | + | NA | + | + | + | Weiss (2016) | |
|  | c.3008G>A/p.G1003D | De novo | ATPase-HELICc | Intellectual disability | | + | | + | NA | + | + | + | Weiss (2016) | |
|  | c.3033_3034​insCTGAATGTG; ​p.L1009_V1011dup | NA | ATPase-HELICc | Sifrim–Hitz–Weiss syndrome | | NA | | NA | NA | NA | + | NA | Weiss (2016) | |
|  | c.3023_3031del /p.C1012del | De novo | ATPase-HELICc | Congenital heart disease | | NA | | + | NA | NA | + | NA | Sifrim (2016) | |
|  | c.3059A>G/p.N1020S | NA | ATPase-HELICc | Sifrim–Hitz–Weiss syndrome | | + | | + | NA | + | + | - | Weiss (2016) | |
|  | c.3109A>G/p.N1037D | NA | ATPase-HELICc | Neurodevelopmental disorder | | + | | NA | NA | NA | NA | NA | Trinh (2019) | |
|  | c.3181A>C/p.N1061H | NA | HELICc | Neurodevelopmental disorder | | + | | NA | NA | NA | NA | NA | Trinh (2019) | |
|  | c.3203G>A/p.R1068H | De novo  De novo | HELICc | Congenital heart disease  Developmental disorder | | +  NA | | +  + | NA  NA | +  NA | +  NA | NA  NA | Sifrim (2016)  McRae (2017) | |
|  | c.3280 G>A p.E1094K | NA | HELICc | Sifrim–Hitz–Weiss syndrome | | + | | + | NA | - | + | - | Weiss (2016) | |
|  | c.3380G>A/p.R1127Q | De novo  De novo  De novo | HELICc | Intellectual disability/developmental delay  Developmental disorder  Intellectual disability | | +  -  + | | NA  +  + | +  NA  NA | NA  +  + | NA  -  - | NA  +  + | Kosmicki (2017)  McRae (2017)  Weiss (2016) | |
|  | c.3441C>G/p.D1147E | NA | HELICc | Sifrim–Hitz–Weiss syndrome | | NA | | + | NA | NA | + | NA | Weiss (2016) | |
|  | c.3443G>T/p.W1148L | De novo | HELICc | Intellectual disability | | NA | | + | NA | + | + | NA | Weiss (2016) | |
|  | c.3518G>T/p.R1173L | NA  NA | HELICc | Intellectual Disability Syndrome with Distinctive Dysmorphisms  Intellectual disability, macrocephaly, hyperlaxity of finger joints and hearing loss | | +  + | | +  + | NA  NA | +  + | -  - | -  + | Weiss (2016)  Monroe (2016) | |
|  | c.3548G>A/p.R1183H | NA | HELICc | Sifrim–Hitz–Weiss syndrome | | - | | + | NA | + | + | + | Weiss (2016) | |
|  | c.3547C>T/p.R1183C | NA | HELICc | Sifrim–Hitz–Weiss syndrome | | + | | + | NA | + | + | + | Weiss (2016) | |
|  | c.3563C>T/p.A1188V | NA | HELICc | Sifrim–Hitz–Weiss syndrome | | NA | | + | NA | + | + | + | Weiss (2016) | |
|  | c.3575T>G/p.M1192R | NA | HELICc | Sifrim–Hitz–Weiss syndrome | | NA | | + | NA | + | + | - | Weiss (2016) | |
|  | c.3745T>G/p.Y1249D | NA | HELICc- DUF1087 | Sifrim–Hitz–Weiss syndrome | | + | | + | NA | + | + | + | Weiss (2016) | |
|  | c.4018C>T/p.R1340C | NA | DUF1087 | Sifrim–Hitz–Weiss syndrome | | + | | + | NA | + | - | - | Weiss (2016) | |
|  | c.4033T>G/p.Y1345D | De novo  NA  De novo | DUF1087 | Congenital heart disease  Congenital heart disease with neurodevelopmental disability  Congenital heart disease with extracardiac anomalies & Neurodevelopmental disorder | | NA  +  + | | NA  NA  + | NA  NA  NA | NA  NA  - | +  +  + | NA  NA  + | Kosmicki (2017)  Homsy (2015)  Jin (2017) | |
|  | c.4802C>T/p.A1601V | NA | DUF1086- CHDCT2 | Neurodevelopmental disorder | | + | | NA | NA | NA | NA | NA | Trinh (2019) | |
|  | c.4822G>A/p.V1608I | De novo  De novo  NA | DUF1086- CHDCT2 | Congenital heart disease  Developmental disorder  Normal phenotype | | NA  NA  - | | +  +  - | NA  NA  - | NA  NA  - | +  NA  - | NA  NA  - | Sifrim (2016)  McRae (2017)  Fattahi (2019) | |
|  | c.4926G>C/p.E1642D | NA | DUF1086- CHDCT2 | Neurodevelopmental disorder | | + | | NA | NA | NA | NA | NA | Trinh (2019) | |
|  | c.4936G>A/p.E1646K | De novo | DUF1086- CHDCT2 | CAE | | - | | - | + | - | - | - | Present study | |
|  | c.4977C>G/p.D1659E | Father | DUF1086- CHDCT2 | BECTS | | borderline | | - | + | - | + | - | Present study | |
|  | c.5083C>T/p.R1695C | Not maternal | DUF1086- CHDCT2 | Rett syndrome | | NA | | NA | NA | NA | NA | NA | Sajan (2017) | |
|  | c.5149C>T/p.R1717W | Father | DUF1086- CHDCT2 | Pituitary stalk interruption syndrome | | NA | | NA | NA | NA | NA | NA | Zwaveling-Soonawala (2018) | |
|  | c.5608 C>T/p.R1870X | NA | CHDCT2 | Sifrim–Hitz–Weiss syndrome | | NA | | + | NA | NA | - | - | Weiss (2016) | |
|  | c.5707C>T/p.P1903S | NA | CHDCT2-C linker | Neurodevelopmental disorder | | + | | NA | NA | NA | NA | NA | Trinh (2019) | |

**Abbreviations:** NA, not available.
