# Supplementary material for: CHD4 variants are associated with childhood idiopathic epilepsy with sinus arrhythmia
Source: CNS Neurosci Ther. 2021 Jun 9;27(10):1146–56. doi: 10.1111/cns.13692 (PMC8446219; doi:10.1111/cns.13692)
Supplement: Supplementary file 4 — Table S4 [file CNS-27-1146-s001.docx]

**Supplementary table 4. Five Clinical-Genetic Dimensional Evaluation of Epilepsy as a Phenotype of *CHD4* Variants.**

|  | **Epilepsy** |
| --- | --- |
| **1. Repetition:** variants recurrently identified in unrelated cases of homogenous phenotype, or significantly high frequency or hotspot in patients | **Yes.** Identified in 4 unrelated cases with childhood idiopathic epilepsy. The frequency of variant alleles was 0.00415. |
| **2. Genotype-phenotype correlation:** for heterogeneous phenotypes, a phenotype was within the spectrum that is correlated with genotype | **Yes.** Epilepsy-related mutations are all missense mutations, while destructive mutations were all associated with multiple congenital abnormalities. |
| **3. Inheritance pattern:** co-segregation in families with AD/AR inheritance, or de novo origination | **Yes.** AD inheritance pattern. Two mutations were de novo and two mutations were segregated in families. |
| **4. Genetic quantitative correlation:** correlation between genetic impairment and phenotype severity | **Not available.** |
| **5. Molecular sub-regional implications:** sub-regional or sub-molecular effects of genetic variants, or distinct functional alteration/mechanism | **Yes.** The missense mutations located in the regions from SNF2 super family domain to DUF1087 domain were associated with multisystem developmental disorders, while epilepsy-related mutations were outside this area. |
